# Physical Fitness Is Negatively Associated With DNA Methylation‐Based Risk of Aging‐Related Diseases

**DOI:** 10.1111/acel.70467

**Published:** 2026-04-08

**Authors:** Samiha Nasser, Johanna Keringer, Yaodong Gu, Istvan Boldogh, Xueqing Ba, Mitsuru Higuchi, Csaba Kerepesi, Zsolt Radak

**Affiliations:** ^1^ HUN‐REN Institute for Computer Science and Control (SZTAKI) Budapest Hungary; ^2^ Faculty of Informatics Eötvös Loránd University (ELTE) Budapest Hungary; ^3^ Doctoral School, and Research Center for Molecular Exercise Science Hungarian University of Sport Science Budapest Hungary; ^4^ Faculty of Sport Science Ningbo University Ningbo China; ^5^ Department of Microbiology and Immunology University of Texas Medical Branch at Galveston Galveston Texas USA; ^6^ Division of Human Health, Key Laboratory of Molecular Epigenetics of Ministry of Education Northeast Normal University Changchun China; ^7^ Faculty of Sport Sciences Waseda University Tokorozawa Japan; ^8^ HUN‐REN‐SZTAKI‐SU Rejuvenation Research Group HUN‐REN Office for Supported Research Groups (TKI) Budapest Hungary; ^9^ Institute of Sport Sciences and Physical Education, Faculty of Sciences University of Pécs Pécs Hungary

## Abstract

Physical fitness is a key determinant of health, yet the molecular pathways linking fitness to the risk of aging‐related diseases remain unclear. We examined associations between DNA methylation–based protein level estimates (EpiScores) and five fitness traits—VO_2_max, GripStrength, JumpMax, body mass index (BMI), and cognition—in a cohort of 290 mostly old individuals (mean age of 60 ± 11 years). EpiScores for 109 plasma proteins were obtained using the MethylDetectR tool and tested for associations with fitness traits. We found 33 significant fitness predictor–EpiScore associations independent from age and sex. Integration with available EpiScore–disease associations revealed overlapping pathways linking fitness and chronic disease risk. We found 51 fitness predictor–disease associations based on EpiScores. The BMI was positively associated with diabetes, stroke, ischemic heart disease, lung cancer, COPD, IBD, and depression, while showing a negative association with rheumatoid arthritis. Cognition was negatively associated with rheumatoid arthritis, depression, and COPD. GripStrength showed negative associations with diabetes and COPD. Finally, jump performance was negatively associated with diabetes, stroke, lung cancer, rheumatoid arthritis, and COPD. We also developed a workflow for evaluating patient‐level disease risk by using DNA methylation and fitness measurements. The patient‐level risk scores showed strong positive correlations with an independent external CVD EpiScore benchmark supporting validity. Our findings highlight the link between cognitive and physical fitness and protein EpiScores as interpretable molecular markers with potential value for early disease risk stratification and for personalized prevention of aging‐related diseases.

## Graphical abstract

We found 33 significant associations between DNA methylation–based protein level estimates (EpiScores) and fitness traits in older adults. Then we found 51 associations between fitness traits and chronic age‐related diseases, enabling a workflow for evaluating patient‐level disease risk by using DNA methylation and fitness measurements.

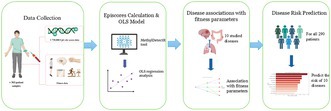

## 1 Introduction

DNA methylation (DNAm) is emerging as a new, personalized tool to predict the risk of various kinds of diseases, and it reflects genetic, lifestyle, and environmental factors (Zhang et al. 2017). Indeed, DNAm can reflect the progress of aging and all‐cause mortality (Lu et al. 2019). Moreover, DNAm‐based epigenetic clocks like PhenoAge acceleration (AgeAccelPheno) and DNAm GrimAge acceleration (AgeAccelGrim) were tested to predict cancer, and the results are auspicious (Li et al. 2022; Dugue et al. 2020).

However, it has been claimed on the results of epigenome‐wide association studies (EWAS) that aging‐associated phenotypes and diseases linked to DNAm could be very different (Jung and Pfeifer 2015). EWAS pointed out that DNAm correlates with C‐reactive protein (Ligthart et al. 2016), and it can predict chronic inflammation, and the methylation score is also associated with cognitive function (Stevenson et al. 2020). We have recently reported that the abundance of pro‐inflammatory bacterial species of *Escherichia coli* and *Collinsella aerofaciens* in the gut microbiome is associated with accelerated DNAm‐based age (Torma et al. 2024). Indeed, DNAm and expressions of genes are altered in various kinds of diseases, including diabetes (Chambers et al. 2015), cardiovascular diseases (Zhong et al. 2016), Alzheimer's disease (Lord and Cruchaga 2014), stroke (Wang et al. 2021), breast cancer (Pashayan et al. 2020), lung cancer (Li et al. 2022), Crohn's disease (Joustra et al. 2025), and obesity (Yaskolka Meir et al. 2025).

Building on these observations, a DNAm‐based circulated proteome was used to calculate EpiScores, which are DNA methylation‐derived estimators of plasma protein levels rather than direct measurements of circulating proteins (Gadd et al. 2022). In the original study, EpiScores were trained for 953 plasma proteins and shown to capture biologically meaningful variation in the circulating proteome, with many scores associated with age‐related diseases, morbidity, and mortality risk (Gadd et al. 2022). Hence, EpiScores could be used as a potential, personalized biological signature that can initiate changes in lifestyle, regular targeted screening, and before disease onset.

**FIGURE 1:**
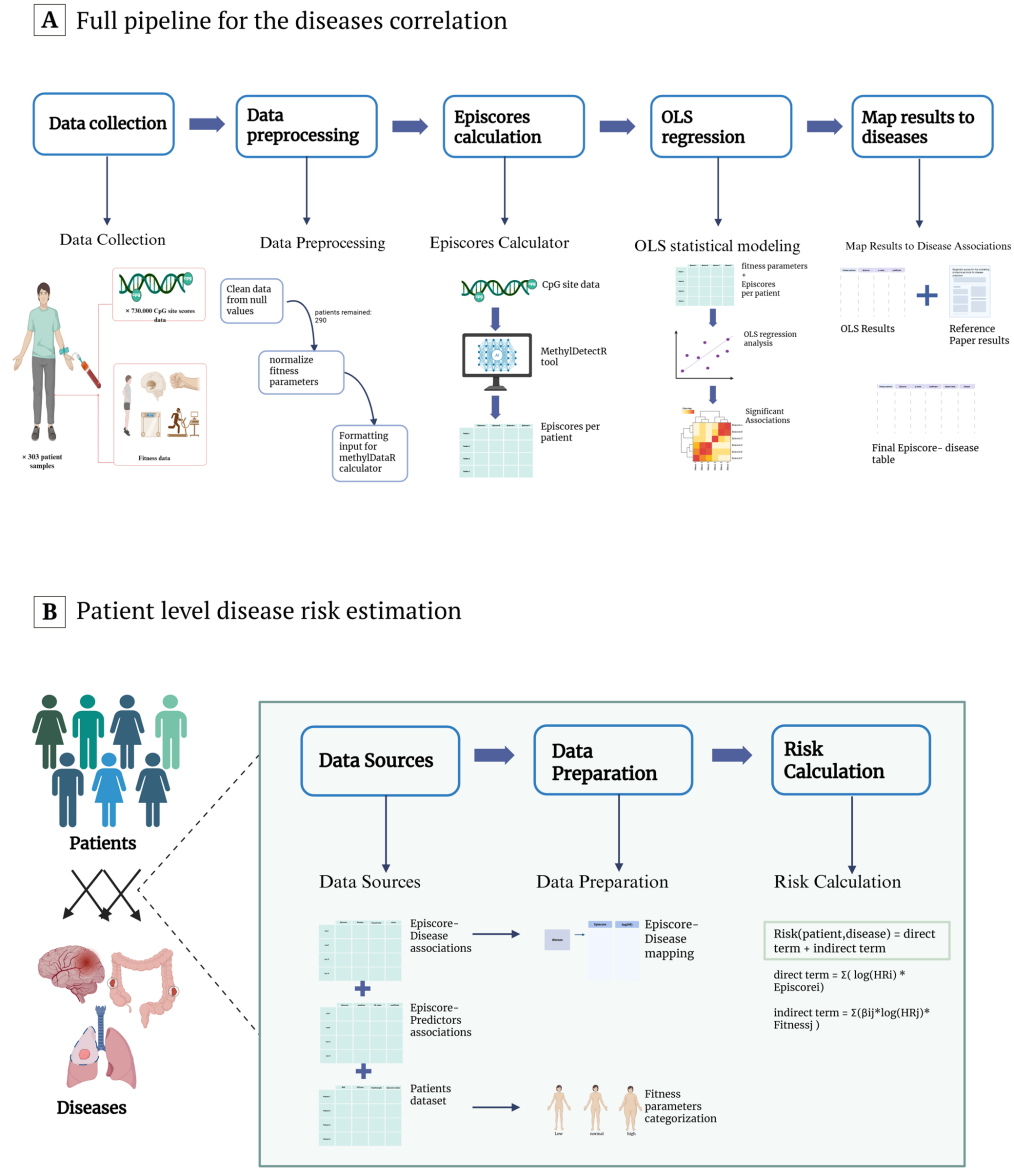
Study workflow. (A) The pipeline of the analysis for determining indirect associations of fitness parameters, EpiScores, and disease risks. (B) The pipeline of patient‐level disease risk prediction.

In addition to DNAm‐based biomarkers, modifiable lifestyle‐related factors such as physical fitness may influence disease risk and biological aging. Regular physical exercise and the level of physical fitness are associated with decreased levels of mortality and decreased incidence of a wide range of lifestyle‐related diseases (Terada et al. 2022; Lang et al. 2024). We have recently shown that a higher level of physical fitness slows down the speed of aging measured by DNAmFitAge (Jokai et al. 2023), and Olympic champions have a decelerated aging process compared to non‐champions, assessed by DNA methylation‐based aging clocks (Radák et al. 2025). A previous study predicted plasma protein levels from DNAm profiles (EpiScores) and associated these scores with disease risk (Gadd et al. 2022). Here, we calculated the plasma protein EpiScores of 290 matched blood methylomes and fitness measurements, and using reported EpiScore–disease associations, we identified the fitness‐associated EpiScores and examined links between fitness and disease risks (Figure 1). To enhance interpretability and robustness, we further evaluated the derived patient‐level risk scores against an independent externally developed CVD EpiScore framework, enabling assessment of validity.

## 2 Results

### 2.1 Description of the Study Cohort

We used a dataset comprised of 290 participants after excluding individuals with missing data in the five fitness predictors (VO_2_max, GripStrength, JumpMax, BMI, and Cognition), DNA methylation profiles, or covariates. The cohort is composed of 52% female and 48% male participants, with a mean age of 60 ± 11 years (range 33–86 years).

### 2.2 Determination of Fitness‐EpiScore Associations

**FIGURE 2:**
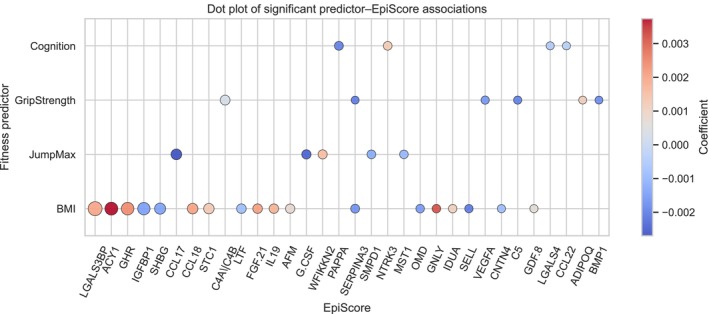
Determination of fitness predictor‐EpiScores associations independent from age and sex. The coefficients (color of the circle) and *p* values (size of the circle) of each OLS model in which we used a given fitness measurement, along with age and sex as covariates, and a plasma protein EpiScore as a target variable. We found 33 significant (*p* < 0.05) fitness predictor–EpiScore associations independent from age and sex.

EpiScores for 109 plasma proteins were obtained using the MethylDetectR tool (Hillary and Marioni 2021) and tested for associations with fitness traits via ordinary least squares regression. Each EpiScore represents a weighted linear combination of methylation beta values corresponding to CpG sites predictive of the concentration of a specific circulating protein. The final dataset comprised a 290 × 117 matrix, with each row representing a participant and the columns containing covariates (age and sex), five fitness‐related variables (VO_2_max, GripStrength, JumpMax, BMI, and cognition), and 109 EpiScores corresponding to predicted protein levels (Table S1). To investigate the relationships between fitness predictors and EpiScores, we used ordinary least squares (OLS) regression models adjusted for age and sex. We found 33 significant (*p* < 0.05) fitness predictor–EpiScore associations independent from age and sex (Figure 2 and Table S2). Because FDR correction substantially reduced the number of significant associations and did not retain all fitness predictors, we presented the full set of nominal associations to illustrate broader fitness–EpiScore trends, while emphasizing FDR‐adjusted results for statistical robustness. FDR‐adjusted significance is reported in Table S3, where a subset of associations remained significant after correction. This analysis highlighted BMI as the fitness trait most frequently associated with EpiScores, contributing to 18 of the 33 significant associations. The JumpMax, GripStrength, and cognition also demonstrated significant associations with EpiScores (i.e., DNA methylation–derived plasma protein levels). Interestingly, VO_2_max was not significantly associated with any EpiScores.

### 2.3 Overlapping Associations Between Fitness Predictors, EpiScores, and Disease Risks

**FIGURE 3:**
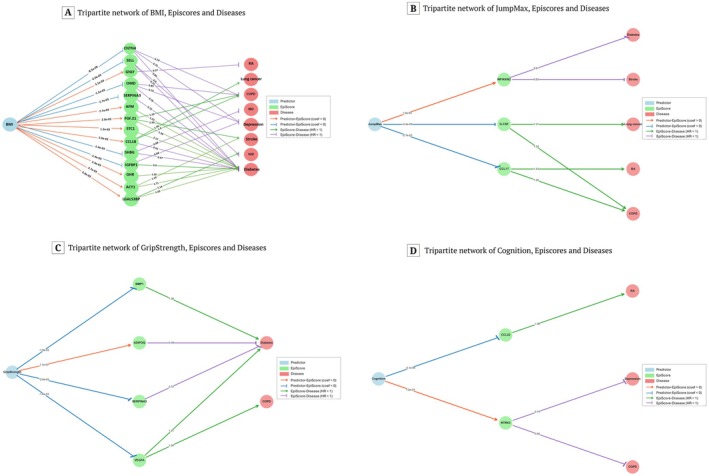
Significant associations between fitness predictors, EpiScores, and diseases. The relationships among fitness predictors, EpiScores (i.e., plasma protein levels predicted from DNA methylation), and disease risk. The coefficients between fitness predictors and EpiScores are from the OLS models calculated by this study. The hazard ratios between protein EpiScores and diseases are from the Episcore‐Disease associations study (Gadd et al. 2022).

By matching the 33 significant fitness predictor–EpiScore associations with previously reported EpiScore–disease associations (Gadd et al. 2022; Chybowska et al. 2024), we found 51 fitness predictor–disease associations (Figure 3 and Table S3). Thirty‐eight of the 51 associations showed consistency in direction with the previously reported EpiScore–disease associations (Gadd et al. 2022). We observed that the direction of associations suggests that BMI‐related EpiScores are positively associated with diabetes, stroke, ischemic heart disease, lung cancer, COPD, IBD, and depression, while showing a surprising negative association with rheumatoid arthritis. Cognition was inversely related to rheumatoid arthritis, depression, and COPD. GripStrength showed negative associations with diabetes and COPD. Finally, jump performance was inversely associated with diabetes, stroke, lung cancer, rheumatoid arthritis, and COPD. In summary, we showed intricate connections between fitness and disease risk determined from DNAm data.

### 2.4 Patient‐Level Disease Risk Estimation

We calculated cohort‐relative disease risk scores for 290 patients across 10 diseases. The risk scores integrated direct effects of EpiScores and indirect effects of fitness predictors (Figure 1B, also see the Methods) and reflect relative risk ranking within the analyzed cohort rather than calibrated clinical risk. The final output consisted of a table of continuous disease risk scores and binary indicators for disease risk classification (Table S4). Each row in the table includes: (i) continuous risk score estimates for 10 diseases, (ii) fitness status classification as low, normal, or high‐risk (based on the literature), and (iii) binary flags (TRUE or FALSE) indicating whether the patient exceeded the disease‐specific high‐risk threshold.

**FIGURE 4:**
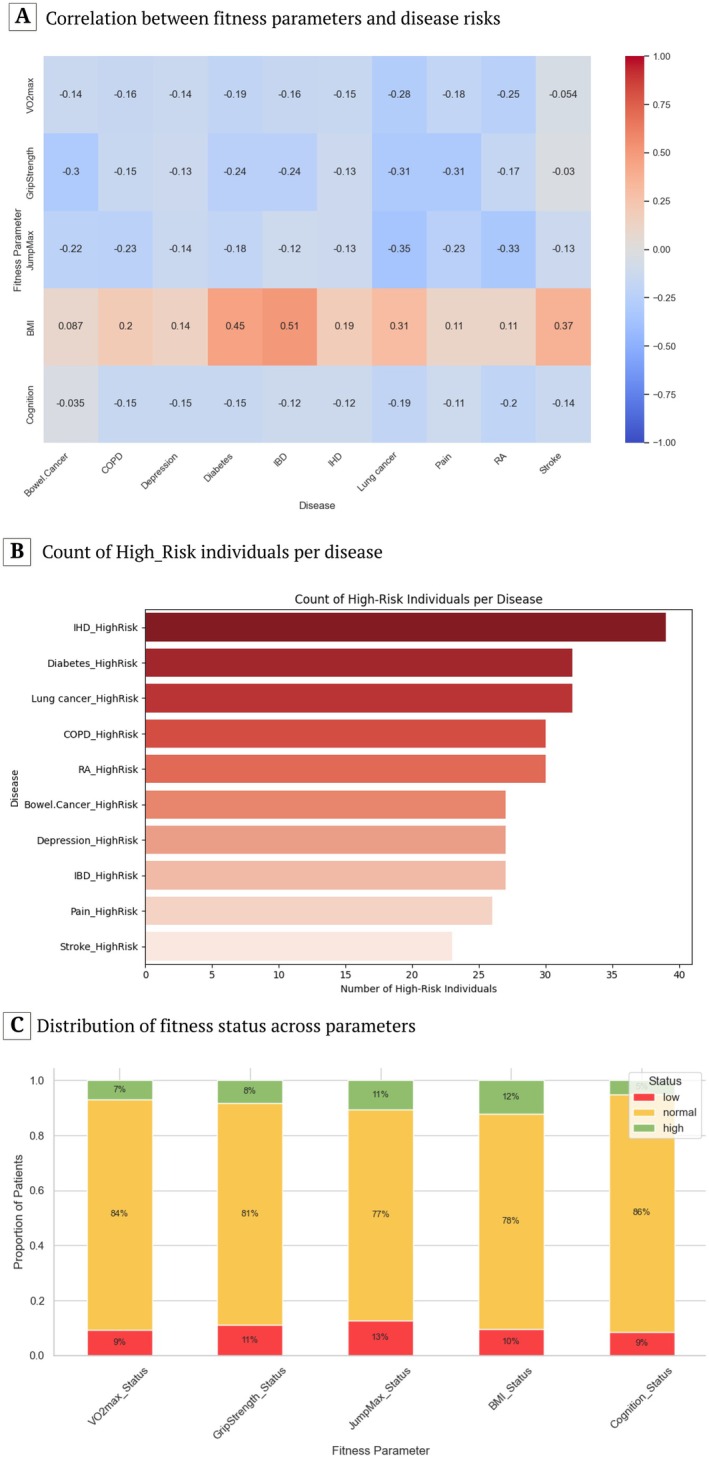
Patient‐level disease risk estimation. (A) Pairwise Pearson correlation between fitness traits (VO_2_max, grip strength, jump maximum, BMI, and cognition) and binary indicators of disease status (bowel cancer, COPD, depression, diabetes, IBD, IHD, lung cancer, pain, RA, and stroke). Values represent correlation coefficients (−1 to +1), with positive correlations in red and negative in blue. (B) Counts of patients classified as high risk for each of 10 diseases (*N* = 290). (C) Stacked barplot showing proportions of patients in low, normal, and high categories for five fitness predictors (VO_2_max, GripStrength, JumpMax, BMI, and Cognition).

To examine the linear associations between fitness traits and predicted disease risk scores, we computed pairwise Pearson correlation coefficients between each fitness parameter and disease‐specific risk estimate (Figure 4A and Figure S2). JumpMax was negatively correlated with RA risk (*r* = −0.33), suggesting that impaired lower‐body strength or neuromuscular function may contribute to higher RA risk. BMI showed positive correlations with diabetes and IBD risks (*r* = 0.45 and *r* = 0.51, respectively), supporting its role as a key metabolic risk factor. Furthermore, a clear negative trend was observed between VO_2_max and COPD risk, reinforcing the protective role of higher aerobic capacity. We also observed that higher BMI was associated with increased diabetes risk, consistent with metabolic disease pathways.

In our cohort, 42.76% of patients were flagged as high‐risk for at least one disease, and the most frequently flagged diseases were IHD, diabetes, and lung cancer, while IBD, pain, and stroke showed the fewest high‐risk predictions (Figure 4B). Of the patients, 9% had low VO_2_max, 78% had normal BMI, and 5% had high cognition. This suggests a generally average aerobic capacity across the cohort. Conversely, GripStrength and JumpMax showed balanced distributions, with 80% of patients in the “normal” range, aligning with population norms.

**FIGURE 5:**
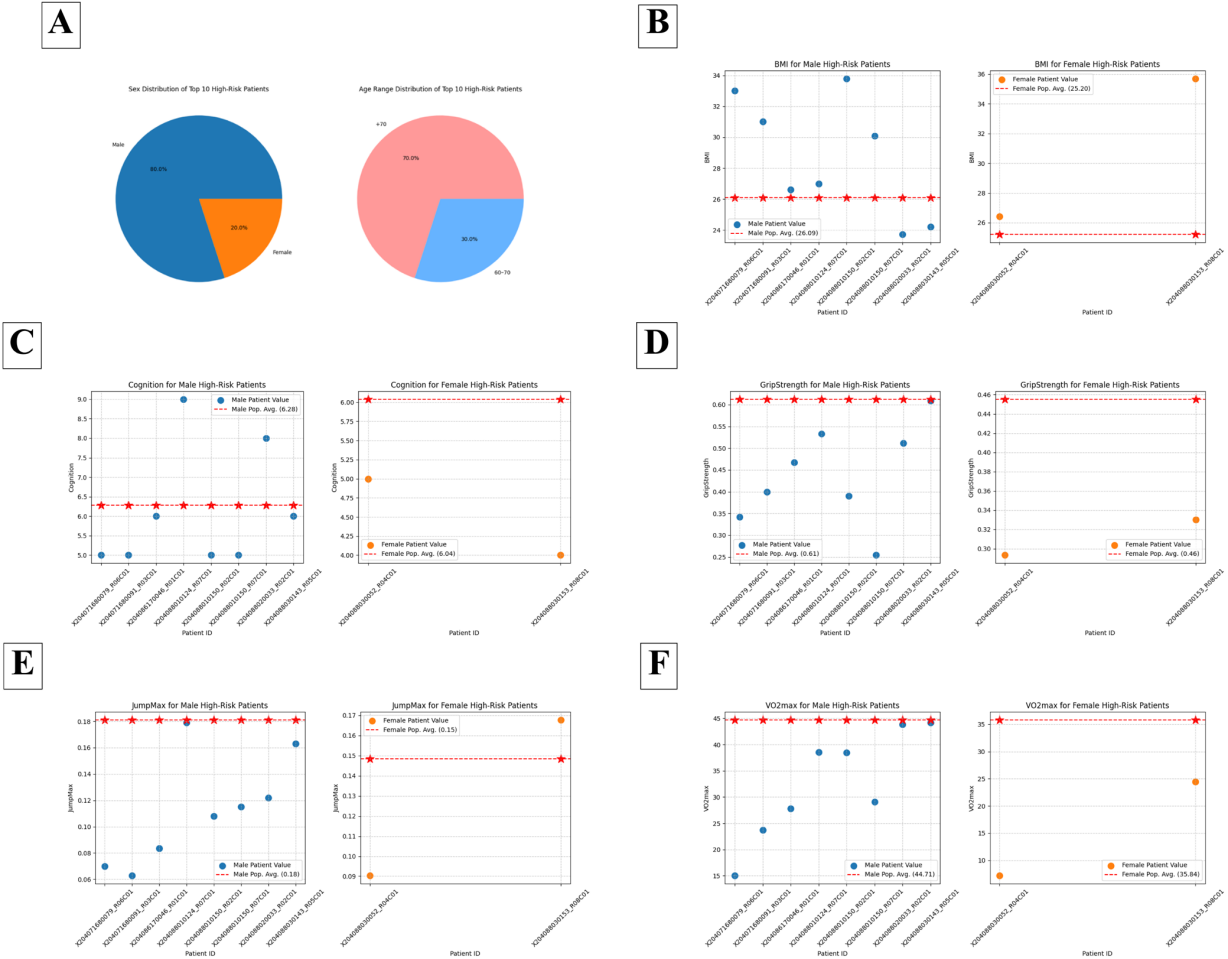
The top 10 patients with the highest number of flagged diseases. (A) Age range and sex distribution of the top 10 patients with the highest number of flagged diseases. (B–F) Individual fitness values for the top 10 patients by sex (points) with sex‐specific population means indicated (dashed line and star).

A total of 10 patients with the highest number of high‐risk disease classifications were identified (Figure 5 and Table S5). The number of flagged diseases per patient ranged from 6 to 9. The most frequently flagged conditions among this group included COPD and IHD, suggesting a clustering of specific disease risks in certain individuals. The majority of these high‐risk patients fell within the (60–70, +70) age range. The top 10 high‐risk patients comprised 80% males and 20% females.

### 2.5 Validation of Risk Scores Against External CVD Benchmarks

**FIGURE 6:**
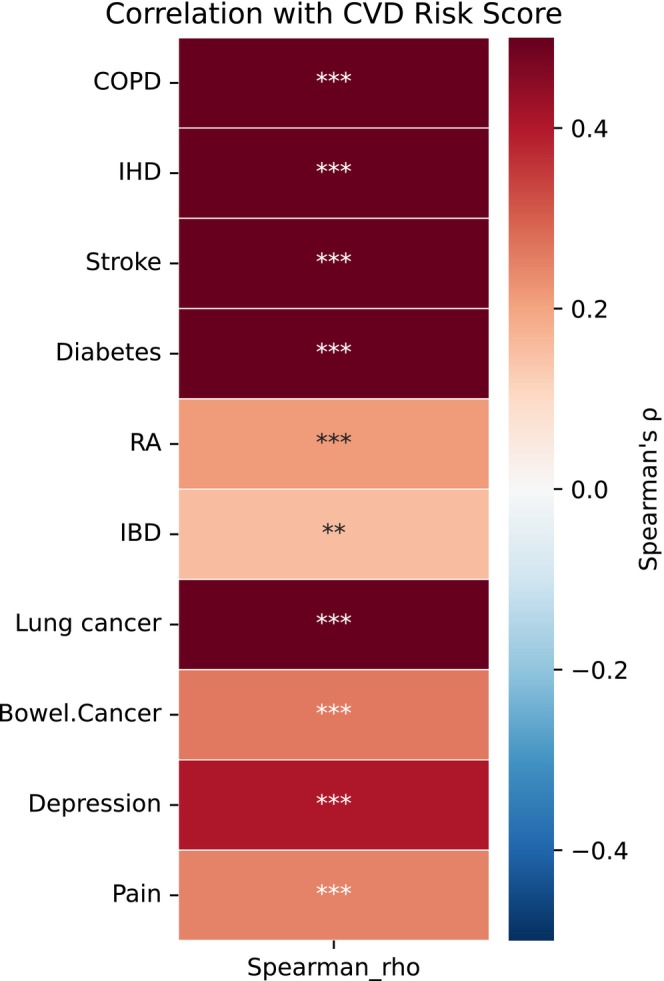
Spearman correlations between CVD risk score and disease‐specific risk score. *p* Values were adjusted for multiple testing using the Benjamini–Hochberg false discovery rate (FDR) method. Color intensity reflects the magnitude and direction of the correlation (red = positive, blue = negative), and significance is indicated by asterisks (**FDR < 0.01, ***FDR < 0.001). Strong positive correlations were observed for COPD, IHD, stroke, diabetes, and lung cancer.

The external CVD risk score, calculated using the 45 protein EpiScores and corresponding weights from the independent study (Chybowska et al. 2024) (Table S8), showed highly significant positive correlations (FDR‐adjusted ***p < 0.001) with all 10 patient‐level disease risk scores examined (bowel cancer, COPD, depression, diabetes, IBD, IHD, lung cancer, pain, RA, and stroke). Correlation coefficients were strongest for lung cancer (*ρ* = 0.85), stroke (*ρ* = 0.62), COPD (*ρ* = 0.58), diabetes (*ρ* = 0.55), and IHD (*ρ* = 0.54) (Figure 6 and Table S9). Correlations exceeded *ρ* = 0.50 for IHD, COPD, diabetes, and stroke, while remaining significant but more modest for conditions such as bowel cancer (*ρ* = 0.26) and IBD (*ρ* = 0.16). These patterns are consistent with shared biological pathways (e.g., inflammation, metabolic dysregulation, and immune response) known to underlie cardiometabolic, respiratory, and related conditions, supporting the convergent validity of our fitness‐mediated risk estimation framework despite the modest cohort size, The overall correlation structure between fitness traits, cognition, demographic variables, and the external CVD risk score is illustrated in Figure S3, demonstrating inverse associations between the CVD score and fitness‐related parameters and positive associations with BMI and age.

## 3 Discussion

We used DNA methylation‐based protein level estimates (EpiScores) to predict the incidence of diseases and examined the relation to physical fitness‐related markers, such as BMI, VO2max, GripStrength, JumpMax, and Cognition. Our data analysis revealed that BMI is one of the most potent predictors of diseases. We found that BMI was positively associated with lung cancer, COPD, IBD, depression, stroke, IHD, and diabetes. It is estimated that heritability rates for BMI range from 40% to 70%, and the association between obesity (high BMI) and a broader range of diseases, such as type 2 diabetes, inflammation, metabolic, cardiovascular, and mental diseases (Chybowska et al. 2024) is well‐documented (Stefan and Schulze 2023; Keller et al. 2023; Ma and Kang 2019). When the relationships between BMI, educational level, diet, physical activity levels, and DunedinPace scores were investigated, BMI showed the strongest association with this DNAm‐based clock in the Multiethnic Cohort study among individuals aged 45–76 years (Maunakea et al. 2024). Similar results have been published in a Finnish Twin Cohort study, which reported a significant association between BMI, insulin resistance, and age acceleration based on GrimAge (Lundgren et al. 2022). Indeed, it was found that changes in adiposity (evaluated by BMI) were associated with DNA methylation at 187 genetic loci, which may link adiposity to metabolic and cardiovascular disease, asthma, T2D, and a wide range of cancers (Wahl et al. 2017). It appears that DNA methylation markers may help distinguish metabolically unhealthy obesity and can be used as an essential tool in personalized medicine (Gutiérrez‐Repiso et al. 2021). Our data confirmed the earlier observation that methylation of the CpG loci encoding LGALS3BP, which are associated with waist circumference (Dhana et al. 2018), could be a risk factor for metabolism and inflammation‐related diseases (Dhana et al. 2018).

It has been shown that a low level of gripping force is associated with sarcopenia via the methylation of Fibroblast growth factor 2 (FGF2) (Li et al. 2024). Here, through EpiScore, we linked GripStrength with diabetes by Bone Morphogenetic Protein 1 (BMP1), adiponectin (ADIPOQ), the serine protease inhibitor, and PDGF/VEGF growth factor family (VEGFA). BMP regulates sugar transporters (Lee et al. 2018), and this could be how greater muscle mass and strength link to GripStrength as a potential diagnostic tool for diabetes.

Our analysis revealed negative associations of GripStrength with diabetes and COPD. There are reports that GripStrength is reduced or maintained in patients suffering from COPD (Cortopassi et al. 2015; Heijdra et al. 2003); however, our data suggest that a low level of GripStrength via VEGFA may be a predictive factor of COPD. We found that GripStrength was associated with the adipokine adiponectin, a key regulator of glucose and lipid metabolism and a known predictor of diabetes risk.

We found that JumpMax was negatively associated with diabetes, stroke, lung cancer, RA, and COPD. We linked JumpMax to WFIKKN2; this protein family has been described as myostatin and growth and differentiation factor 11 inhibitors in muscle and skeletal tissues (Monestier and Blanquet 2016), and now they have emerged as a potential JumpMax‐related predictor of stroke and diabetes. JumpMax also links the glycoprotein Granulocyte colony‐stimulating factor (G‐CSF) that stimulates the bone marrow to produce granulocytes and stem cells, and the survival, proliferation, differentiation, and function of neutrophil precursors and mature neutrophils to the prediction of lung cancer and COPD (Deotare et al. 2015). JumpMax also links the chemokine ligand 17 to the prediction of rheumatic arthritis. Our analysis also showed that cognition is negatively associated with RA, depression, and COPD. Cognition tests via chemokine ligand 22 are also linked to RA (Basile et al. 2021). The cognition test links the TrkC too, which is the catalytic receptor for the neurotrophin NT‐3, as a potential predictor of COPD and depression (Basile et al. 2021).

In this investigation, we utilized physical fitness‐related markers as potential predictors of certain diseases, based on EpiScores, which were derived from DNA methylation. Interestingly enough, among BMI, JumpMax, GripStrength, cognitive test, and VO_2_max, the latter showed no predictive potential, and the BMI and JumpMax showed the highest. EpiScore data on BMI, JumpMax, GripStrength, and cognitive test results align with the findings of epidemiological studies, which report the preventive benefits of these fitness markers. The VO_2_max depends on cardiac output and arterio‐venous oxygen difference (red blood cell levels, capillarization, mitochondrial mass and function) and is divided by body weight. It has been proven to be one of the best markers of mortality and disease risk (Perry et al. 2024). The fact that DNA methylation‐based Episcores could not detect a VO_2_max‐dependent disease association may highlight a limitation of Episcore, since it is built to predict diseases at the level of a single protein. Due to the complex nature of VO_2_max, both cardiac and peripheral, the levels result from multiorgan function, likely affecting hundreds of genes to execute protective effects against diseases.

Physical fitness tests can be used as a predictor of diseases by the DNA methylation‐based EpiScore. On an individual level, EpiScore can serve as a personalized predictor of diseases. It is suggested that subjects who show high risk based on EpiScore with normal or relatively high levels of physiological test results have genetically encoded sensitivity. Conversely, subjects with high risk but low levels of physiological test results have lifestyle‐related, and therefore modifiable, sensitivity.

The highly significant correlations (FDR‐adjusted *p* < 0.001) between our patient‐level disease risk scores and the independently derived external CVD risk score (based on 45 protein‐linked EpiScores and weights from a reference paper) (Chybowska et al. 2024) across all 10 examined diseases provide supportive convergent evidence for biological consistency. Strongest associations were observed for COPD, IHD, stroke, diabetes, and lung cancer—conditions with established cardiometabolic and inflammatory overlap with CVD—while significant but weaker correlations extended to others (bowel cancer, IBD, RA, depression, and pain). This pattern aligns with known pleiotropy in epigenetic biomarkers, where DNA methylation and protein‐derived EpiScores often capture shared upstream mechanisms such as chronic low‐grade inflammation, metabolic dysregulation, and immune pathways that contribute to multiple age‐related and cardiometabolic diseases, rather than isolated CVD specificity (Alhuneafat et al. 2025). For instance, DNA methylation signatures of chronic low‐grade inflammation (e.g., driven by CRP) have been linked to increased risk across cardiometabolic diseases and COPD through overlapping inflammatory and metabolic pathways (Wielscher et al. 2022).

## 4 Methods

### 4.1 Study Design and Participants

We used the DNA methylation profiles from the Hungarian Olympic Champion, a population‐based dataset comprising 388 individuals aged 24–101 years (the mean age is 58 years). DNA methylation was measured using Illumina Infinium MethylationEPIC BeadChip arrays across multiple batches. The MET2019, MET2020, and MET2022 batches used the EPIC v1.0 array, while the MET2023 batch used the EPIC v2.0 array. The v2.0 data were converted to v1 format for batch merging. Full details of sample collection, processing, quality control, and data availability are provided at the NCBI Gene Expression Omnibus (GEO) under accession number GSE295450 (Radák et al. 2025). For the present analysis, we included only participants with complete data (i.e., no missing values) for five fitness‐related variables—VO_2_max, GripStrength, JumpMax, BMI, and cognition—as well as DNA methylation and relevant covariates (age and sex). So, we did not use the data of the Olympic champions as they had no fitness measurements. Instead, we used only the controls of the study. After excluding individuals with missing values in any of these variables, the final sample consisted of 290 participants. The National Public Health Center approved the study in accordance with the Helsinki Declaration and the regulations applicable in Hungary (25167‐6/2019/EÜIG). All participants provided written informed consent prior to inclusion.

### 4.2 Fitness and Phenotypic Measures

Five fitness‐related and cognitive traits were selected as predictors due to their established associations with physical health and aging outcomes: (i) VO_2_max (Estimated Maximal Aerobic Capacity) that is an estimate of relative aerobic capacity based on performance in the Cooper test (participants who were unable to complete the test were classified as “low aerobic capacity” and assigned a score of 0, following the VO_2_max classification guidelines in the Cooper reference table), (ii) GripStrength [kg/BM] (Grip Strength Normalized by Body Mass) that is the maximum grip strength (in kilograms) measured using a hand dynamometer, normalized by the participant's body weight (kg/body mass), (iii) JumpMax [cm/BH] (Vertical Jump Height Normalized by Body Height) that is the maximum vertical jump height (in centimeters), normalized by standing body height (cm/body height), used as a relative strength and power indicator, (iv) BMI (Body Mass Index) that is a standard metric of weight status, calculated as weight in kilograms divided by height in meters squared (kg/m^2^), and (v) Cognition (Verbal Short‐Term Memory) that measures a cognitive performance score derived from a digit span test, which assesses verbal short‐term memory capacity. All predictor variables were normalized using z‐score transformation prior to regression modeling (Table S6).

### 4.3 EpiScore Calculation

We calculated EpiScores for 109 circulating proteins using the MethylDetectR framework (Hillary and Marioni 2021). The tool is based on publicly available CpG weights from a previous study (Gadd et al. 2022). Each EpiScore represents a weighted sum of methylation beta values and serves as a proxy estimate for the corresponding plasma protein concentration. We followed the command‐line workflow recommended by the original authors, using scripts and resources from the companion repository (Hillary 2020). The following input files were used: (i) methylation data in .rds format, (ii) age and sex covariates in .csv format, (iii) Predictors_Shiny_by_Groups.csv, (iv) coefficient files: cage_coefficients_linear.tsv, cage_coefficients_log.tsv, and (v) CpG mean reference: cpg_meanbeta_gs20k.tsv. Two core scripts were run: (i) convert_to_rds.R—to format raw data (where the methylation matrix was transposed to have CpG sites as rows and sample IDs as columns)—and (ii) Script_For_User_To_Generate_Scores.R—to calculate EpiScores. This allowed efficient generation of EpiScores for local computation without relying on the Shiny front‐end.

### 4.4 EpiScore Name Mapping

EpiScore names generated by MethylDetectR (Hillary and Marioni 2021) were manually mapped to the standardized nomenclature used in a large EpiScore–disease study (Gadd et al. 2022). The goal of this mapping is to align different naming conventions used across platforms and studies and to ensure compatibility with published disease associations. The mapping was performed using the supplementary tables of the same study (Gadd et al. 2022), including (i) annotations for the 109 EpiScores with identifiers (SOMAscan SeqIds or Olink names), gene names (used for SOMAscan EpiScores), and protein targets. It also lists CpG counts for each EpiScore, which were cross‐referenced with the *MethylDetectR* tool repository (Hillary 2020) to validate mappings. For example, the MethylDetectR EpiScore “ADAMTS” was mapped to “ADAMTS13” (Identifier: 3175‐51, SOMAscan). All mappings were confirmed as correct by the original authors of the MethylDetectR study. The mappings are available in Table S7, which includes MethylDetectR EpiScore names (EpiScore_Name) and standardized names from EpiScore–disease study (Gadd et al. 2022) (Standard_Name), identifiers, assay panels (SOMAscan or Olink), and notes on duplicates (e.g., “CLEC11A e1” and “CLEC11A e2” mapping to “CLEC11A”). This harmonization step was essential to ensure that disease associations were linked to the correct protein targets across platforms and studies, thereby avoiding misinterpretation arising from naming inconsistencies.

### 4.5 Associations Between Fitness Predictors, EpiScores, and Diseases

Ordinary least squares (OLS) regression models were used to examine associations between each fitness predictor and each EpiScore. For every model, EpiScores were treated as dependent variables, with fitness predictors as independent variables and age and sex included as covariates. The sex variable was numerically coded (female = 0, male = 1). Additional adjustment for other factors (e.g., cell‐type composition or lifestyle variables) was not performed due to the focus on extreme athletic phenotypes and limited availability of additional covariates in controls. The general model formula was:

Episcore∼Fitness Predictor+Sex+Age

Statistical significance was initially determined using a nominal *p* value threshold of *p* < 0.05 to identify candidate fitness–EpiScore associations. Given the exploratory nature of this analysis and the large number of regressions performed, false discovery rate (FDR) correction was additionally applied and reported in Table S3 to control for multiple testing. To evaluate the biological relevance of identified predictor–EpiScore associations, we compared our results with previously published hazard ratios for EpiScore–disease relationships reported in EpiScore–disease study (Gadd et al. 2022). For each significant predictor–EpiScore pair, we assessed whether the sign of the regression coefficient matched the direction of the published disease hazard ratio (HR > 1 indicating increased risk, HR < 1 indicating reduced risk). Logical consistency was determined by evaluating the causal chain: (i) Predictor → EpiScore (our model), (ii) EpiScore → Disease (published HR).

### 4.6 Fitness Predictor Thresholds and Categorization

Thresholds for fitness predictors (VO_2_max, GripStrength, JumpMax, BMI, and Cognition) were established using age‐ and sex‐specific norms from the literature to categorize patient performance as “low,” “normal,” or “high.” For VO_2_max, GripStrength, JumpMax, and BMI, thresholds were set at ±1.28 standard deviations (SD) from the mean for specific age groups (e.g., 50–60 and 60–70 years), based on established guidelines (Liguori et al. 2014). For Cognition, thresholds were defined at the 10th and 90th percentiles of patient data to identify impaired and exceptional performance (Lezak et al. 2012). Patient values were compared to these thresholds using Python functions, assigning categories such that, for example, a VO_2_max value below −1.28 SD was “low,” above +1.28 SD was “high,” and otherwise “normal.” This approach aligns with clinical risk stratification frameworks.

### 4.7 Patient‐Level Disease Risk Scores Calculation

The patient‐level disease risk scores integrate both the direct effects of EpiScores on disease risk and the indirect effects derived from fitness–EpiScore associations. As risk scores were standardized to *z*‐scores based on the study dataset's distribution, they represent relative risk estimates within the analyzed cohort. To quantify the direct effects of EpiScores on disease risk, we extracted the natural logarithm of hazard ratios log(HR) from the Episcore‐disease study (Gadd et al. 2022). In that study, associations were assessed using Cox proportional hazards models; basic models adjusted for age and sex, while fully adjusted models additionally accounted for common risk factors including smoking status, social deprivation, educational attainment, body mass index (BMI), and alcohol consumption (where available). We applied the reported log(HR) coefficients (derived from the fully adjusted models) as fixed weights to the corresponding EpiScores in our cohort to compute direct disease contributions, without re‐estimating associations in the present dataset. The data were grouped by disease to create a mapping dictionary, linking each disease to its associated EpiScores and their corresponding log (HR) values. Our approach extends the product‐of‐coefficients framework described by a previous study (Zhou et al. 2020), which estimates indirect effects through high‐dimensional mediators. Specifically, we calculated (i) the direct effect of EpiScores on disease risk (using log‐transformed hazard ratios) and (ii) the indirect effect of fitness predictors via EpiScores (using the product of fitness–EpiScore and EpiScore–disease coefficients). This two‐part structure enabled us to capture both biological layers of influence in disease risk estimation, as expressed by the following equation:

$$\begin{array}{c} \text{Risk}\left(\text{patient},\text{disease}\right)=\Sigma \left(\;\log\left({\mathrm{HR}}_{i}\right)\times {\text{Episcore}}_{i}\right)\\ +\Sigma \left(\beta_{i\to j}\times \log\left({\mathrm{HR}}_{j}\right)\times {\text{Fitness}}_{j}\;\right) \end{array}$$

The direct effect term $\Sigma \left(\;\log\left({\mathrm{HR}}_{i}\right)\times {\text{Episcore}}_{i}\right)$ represents a weighted sum of standardized EpiScore values, where the weights are log hazard ratios (HRs) from the EpiScore disease study dataset (Gadd et al. 2022), following the polygenic risk score (PRS) methodology (Choi et al. 2020) The indirect effect term $\Sigma \left(\beta_{i\to j}\times \log\left({\mathrm{HR}}_{j}\right)\times {\text{Fitness}}_{j}\;\right)$ captures the association between fitness predictors and EpiScores: regression coefficients $\beta_{i\to j}$ quantify the influence of each fitness trait (*j*) on disease‐linked EpiScores (*i*), and these effects are propagated through the EpiScore–disease hazard ratios. This two‐part framework separates (i) the direct molecular contribution of EpiScores to disease risk from (ii) the indirect contribution of upstream fitness traits acting through EpiScores, in line with high‐dimensional mediation models (Zhou et al. 2020).

### 4.8 Risk Score Normalization and High‐Risk Identification

To enable comparison across diseases, risk scores were standardized to *z*‐scores based on the study dataset's distribution, reflecting relative risk within this population. Patients with *z*‐scores exceeding 1.28 (approximately the 90th percentile of the dataset) were flagged as high‐risk, identifying individuals in the top 10% of risk for each disease (Khera et al. 2018). This percentile‐based threshold, dependent on the dataset's risk distribution, is commonly used in risk prediction studies to prioritize clinical interventions. The results are available in Table S4.

### 4.9 Identifying the Top 10 High‐Risk Patients Based on Disease Flags

The top 10 patients with the highest number of flagged diseases were selected for further analysis. We generated an output table listing patient IDs alongside the names of diseases for which they were flagged as high risk. This table is available in Table S5.

### 4.10 Validation of Disease Risk Scores Using External CVD Weights

To address potential overfitting in the fitness–EpiScore associations derived from our cohort and to provide a form of benchmarking against external data, we calculated an independent cardiovascular disease (CVD) risk score using weights from an external reference study (Chybowska et al. 2024), For each participant, we subsetted to the 45 EpiScores listed in table IV of the supplemental material from that study. These EpiScores were multiplied by the corresponding external weights to generate a continuous CVD risk score, where higher values indicate higher epigenetic CVD risk:

$$\text{Patient}\;\mathrm{CVD}\;\text{risk score}=\sum_{i=1}^{45}\left({\text{weight}}_{i}\times {\text{Episcore}}_{i}\right)$$

This CVD risk score was then correlated with each of our calculated patient‐level disease risk scores (as defined in the main equation). Pearson correlation coefficients were computed, with *p* values corrected for multiple testing using the false discovery rate (FDR) method.

### 4.11 Limitations

This study has several limitations that should be considered when interpreting the findings. First, the analyses are based on a cross‐sectional design, which limits causal inference between fitness traits, EpiScores, and disease risk. Second, the modest sample size (290 samples) may reduce statistical power and limit the generalizability of the results to other populations. Third, disease risk estimation relied on hazard ratios derived from external studies, assuming their applicability to the present cohort, which may introduce uncertainty related to population differences. Fourth, the analyses were based on DNA methylation–predicted protein EpiScores rather than directly measured circulating protein levels. Finally, due to the lack of an appropriate external validation cohort, direct external validation of the derived disease risk scores was not possible.

## Author Contributions

S.N.: data analysis and statistics, investigation, and writing – review and editing. J.K.: investigation and methodology. Y.G.: investigation, formal analysis, software, and writing – review and editing. I.B.: investigation, validation, and writing – review and editing. I.B., X.B., and M.H.: investigation, project administration, supervision, and writing – review and editing. C.K.: data preprocessing, data analysis and statistics, supervision of data analysis and statistics, investigation, and writing – review and editing. Z.R.: conceptualization, funding acquisition, investigation, methodology, resources, supervision, writing – original draft, and writing – review and editing.

## Funding

The study was supported by the European Union project RRF‐2.3.1‐21‐2022‐00004 within the framework of the Artificial Intelligence National Laboratory. CK was also supported by the National Research, Development and Innovation Office—NKFIH (146113), the HUN‐REN (TKCS‐2024/37), and the János Bolyai Research Scholarship of the Hungarian Academy of Sciences. ZR acknowledges support from the National Science and Research Fund (OTKA142192), and HU‐RIZONT‐2025‐00096.

## Conflicts of Interest

The authors declare no conflicts of interest.

## Supporting information


**Figure S1:** Correlation matrix of fitness predictors. Pairwise Pearson correlations were calculated among VO_2_max, Grip strength, Jump maximum, BMI, and Cognition. Values represent correlation coefficients, with positive correlations shown in red and negative in blue (color intensity indicates strength). This figure illustrates the degree of interdependence among the fitness variables used in subsequent analyses.
**Figure S2:** Scatterplots of disease EpiScores versus continuous fitness parameters. For each of 10 diseases and 5 fitness measures (VO_2_max, Grip strength, Jump maximum, BMI, and Cognition), pairwise associations were visualized using scatterplots with fitted linear regression lines (dashed red). Pearson correlation coefficients and *p* values are shown in the plot titles.
**Figure S3:** Correlation matrix of fitness, cognitive, demographic variables, and CVD risk score. Color intensity indicates the strength and direction of the correlations (red = positive, blue = negative). The CVD risk score exhibited negative correlations with fitness and cognition‐related variables (VO_2_max, grip strength, jump performance, and cognition), and positive correlations with BMI and age.

**Table S1:** The results of EpiScore calculation using the methylDetectR framework. Genome‐wide CpG methylation data were used to compute EpiScores for 109 circulating proteins. The sex variable was numerically coded for regression models (female = 0, male = 1).
**Table S2:** Significant associations by fitting OLS model to the predictor‐episcore dataset after adjusting for sex and age. An association is considered significant if *p* value < 0.05.
**Table S3:** The results of matching the 33 significant fitness predictor–EpiScore associations with previously reported EpiScore–disease associations. It shows 51 fitness predictor–disease associations. All associations show the expected direction except three (see red coloring).
**Table S4:** The results of patient‐level disease risk estimation for 290 patients across 10 diseases, and categorizing fitness predictors as low, normal, and high.
**Table S5:** The top 10 patients with the highest number of flagged diseases.
The sex variable was numerically coded for regression models (female = 0, male = 1).
**Table S6:** Definition and statistics of the fitness parameters used in all of the analysis.
**Table S7:** Episcores naming mapping Episcore_Name is from MethylDetectR results used in Hillary et al., Standard_Name is from Gadd et al. (2022).
**Table S8:** CVD risk score for each patient along with the 45 episcores used in the equation.
**Table S9:** The results of applying Spearman correlation between CVD risk score and disease‐specific risk score. The *p* values were adjusted for multiple testing using the Benjamini–Hochberg false discovery rate (FDR) method.

## Data Availability

Data and codes generated by the study are available in the Tables S1–S9 as well as in the Zenodo public repository (https://zenodo.org/records/19130741).
