# Supplementary material for: Physical Fitness Is Negatively Associated With DNA Methylation‐Based Risk of Aging‐Related Diseases
Source: Aging Cell. 2026 Apr 8;25(4):e70467. doi: 10.1111/acel.70467 (PMC13061595; doi:10.1111/acel.70467)
Supplement: Supplementary file 1 — Figure S1: Correlation matrix of fitness predictors. Pairwise Pearson correlations were calculated among VO2max, Grip strength, Jump maximum, BMI, and Cognition. Values represent correlation coefficients, with positive correlations shown in red and negative in blue (color intensity indicates strength). This figure illustrates the degree of interdependence among the fitness variables used in subsequent analyses. Figure S2: Scatterplots of disease EpiScores versus continuous fitness parameters. For each of 10 diseases and 5 fitness measures (VO2max, Grip strength, Jump maximum, BMI, and Cognition), pairwise associations were visualized using scatterplots with fitted linear regression lines (dashed red). Pearson correlation coefficients and p values are shown in the plot titles. Figure S3: Correlation matrix of fitness, cognitive, demographic variables, and CVD risk score. Color intensity indicates the strength and direction of the correlations (red = positive, blue = negative). The CVD risk score exhibited negative correlations with fitness and cognition‐related variables (VO2max, grip strength, jump performance, and cognition), and positive correlations with BMI and age. [file ACEL-25-e70467-s001.docx]

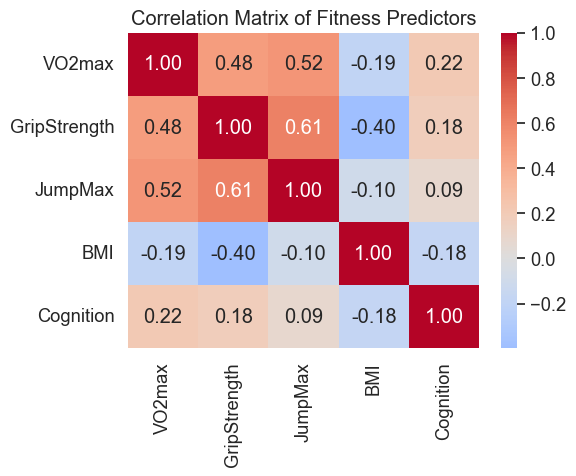


**Supplementary Fig. 1 |** Correlation matrix of fitness predictors. Pairwise Pearson correlations were calculated among VO₂max, Grip strength, Jump maximum, BMI, and Cognition. Values represent correlation coefficients, with positive correlations shown in red and negative in blue (color intensity indicates strength). This figure illustrates the degree of interdependence among the fitness variables used in subsequent analyses.





**Supplementary Fig. 2** | Scatterplots of disease EpiScores versus continuous fitness parameters. For each of 10 diseases and 5 fitness measures (VO₂max, Grip strength, Jump maximum, BMI, Cognition), pairwise associations were visualized using scatterplots with fitted linear regression lines (dashed red). Pearson correlation coefficients and p-values are shown in the plot titles.


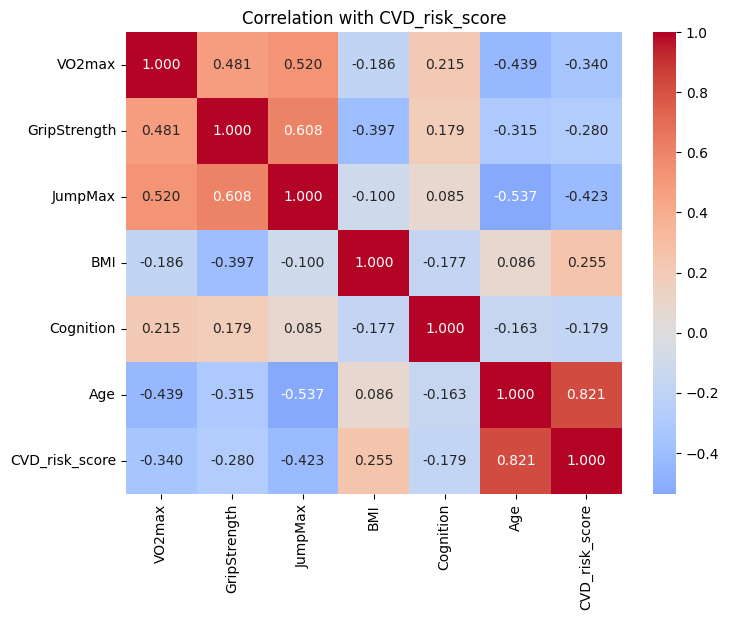


**Supplementary Fig. 3 |** Correlation matrix of fitness, cognitive, demographic variables, and CVD risk score. Color intensity indicates the strength and direction of the correlations (red = positive, blue = negative). The CVD risk score exhibited negative correlations with fitness and cognition-related variables (VO₂max, grip strength, jump performance, and cognition), and positive correlations with BMI and age.
